# Preventing Free Flap Complications: Key Factors to Keep in Mind

**DOI:** 10.1002/jso.70067

**Published:** 2025-08-27

**Authors:** Jonas Kornmann, Rasmus Schug, Lena Huber, Anne Lammert, Frederic Jungbauer, Annette Affolter, Nicole Rotter, Lena Zaubitzer, Luis Bugia, Grietje Beck, Claudia Scherl

**Affiliations:** ^1^ Department of Otorhinolaryngology, Head and Neck Surgery, Medical Faculty Mannheim Heidelberg University Mannheim Germany; ^2^ Department of Anaesthesiology, Medical Faculty Mannheim Heidelberg University Mannheim Germany; ^3^ AI Health Innovation Cluster, Heidelberg‐Mannheim Health and Life Science Alliance Heidelberg Germany

**Keywords:** complications, free flap, head and neck surgery, heparin, perioperative medication, postoperative care, preoperative preparation

## Abstract

**Background and Objectives:**

Reconstruction of head and neck defects using free flaps is successful, but complications occur. This study aims to identify factors preventing complications to support clinical decision‐making.

**Methods:**

Retrospective study for free flap reconstructions (2019 to 2022, tertiary referral center). Univariate and multivariate regression models assessed predictors of complication‐free survival (CFS) and odds ratios (OR) measured risk correlations.

**Results:**

Of 125 identified cases, most patients were male (71.8%) with a median age of 66 years (37–93 years). Common complications were wound healing disorders (10.9%), hematoma (10%), total (7.3%) or partial (1.8%) flap necrosis, cardiovascular events (5.5%), and pulmonary artery embolism (4.5%). 30‐day CFS was 63%. On multivariable analysis, female gender (HR: 9.4, CI: 2.6–33.5), alcohol abuse (HR: 3.5, CI: 1.4–8.4), N2‐3 (HR: 2.4, CI: 1.3–4.4), obesity (HR: 2.1, CI: 0.9–5.1), preoperative anticoagulation (HR: 2.5, CI: 1.1–5.9) were significant prognosticators. Positive factors increasing CFS included high albumin (OR 0.21, *p* = 0.02), intraoperative i.v. heparin bolus (OR 0.15, *p* = 0.08), intraoperative catecholamine treatment (OR 0.15, *p* = 0.009), and nonsmoking (OR 0.18, *p* = 0.1).

**Conclusion:**

Key preventive measures against complications include optimizing nutritional status and albumin levels, administering intraoperative heparin and catecholamines, and abstaining from alcohol. Females should also be screened for undiagnosed cardiovascular risks.

**Abbreviations**
CFS: Complication free survival
PAVD: Peripheral artery disease

## 1 Introduction

Currently, defect coverage using free transplants is the standard therapy for large defects in the head and neck area. Free flap surgery is performed frequently and offers the good rehabilitation for chewing, swallowing, and speaking functions. Despite good surgical expertise and perioperative medical care, complications occur frequently. Presurgical evaluation of medical history, estimated defect size, and reconstructive concepts should be considered to improve patient care and safety [1, 2, 3, 4, 5, 6, 7, 8, 9]. While most published papers focus on the risk factors associated with free flap reconstruction, we emphasize potentially beneficial factors in free flap surgery. We identify body conditions correlated with positive outcomes and discuss intraoperative medications that may help prevent surgical and medical complications. Additionally, we offer valuable recommendations for preoperative preparation and key considerations for postoperative care.

## 2 Material and Methods

This study was approved by the institutional Ethics Committee (#2024‐859). It was conducted in accordance with the Declaration of Helsinki 1975, as revised in 1983. A retrospective review of our tertiary referral centre flap database from 2019 to 2022 was conducted. The retrospective nature of the study did not require informed consent.

### 2.1 Inclusion and Exclusion Criteria

The database initially was searched for all patients who received a flap surgery in the head and neck region. Cases included individuals with free flap surgery, regional and distal pedicled flaps. For this study, patients who did not receive a free flap were ruled out. Cases with missing information on flap outcome were excluded, as information on influencing factors is unknown. This resulted in 110 patients. All included patients received either a radial forearm flap or anterolateral thigh flap.

### 2.2 Study Variables

Patients were grouped and stratified according to gender, age, and TNM classification (according to the American Joint Committee on Cancer classification, Cancer Staging Manual, 8th edition), and defect site. Potential risk factors were grouped as substance abuse, medical premorbidity, body constitution, anticoagulant medication, catecholamine treatment. Age was categorized as < 55 and ≥ 55 years. Defect site was divided into skin and mucosa. Skin includes all external defect coverings on the face and neck, mucosa the areas of the oral cavity, pharynx and larynx. Alcohol and nicotine abuse were considered substance abuse. Medical premorbidity included hypertension, peripheral arterial disease, cardiovascular events (embolia, myocardial infarct, stroke), diabetes mellitus, and albumin levels ≤ 33 and 33‐48 mg/dl. The body constitution was divided into slim and obese. Anticoagulant medication is important and included the investigation of preoperative anticoagulative treatment, intraoperative therapy (Aspirin, Heparinbolus), postoperative fractionated low and high dose heparin.

### 2.3 Prevention of Complications

The primary outcome was complication free survival (CFS). Events and physical changes during and after the inpatient stay are carefully documented by our medical registrars in the database. The database was searched for surgical and medical complications and related to preventative factors and risks.

### 2.4 Statistics

Qualitative variables were described as frequencies and percentages, and differences among groups were tested using Pearson's chi‐square test. The Kaplan‐Meier method was used to depict the complication‐free survival (CFS) at 30 days in life tables and survival curves. The log‐rank test was used to compare the distribution of the groups. Survival calculation refers to the time between the date of surgery and the date of complications. Statistical tests were two‐tailed, and significance was set at *p* ≤ 0.05. Logistic regression was conducted to calculate odds ratios and 95% confidence intervals (CI) to identify factors associated with complications. To determine factors independently associated with increased CFS, a univariate analysis was followed by a multivariable Cox regression analysis. The precision of point estimates is expressed as odds ratios or hazard ratios, accompanied by 95% confidence intervals. The proportional hazard assumption was assessed for validity using the log‐minus‐log plot. SPSS for Windows (version 28, 2022) was used for statistical analyses.

## 3 Results

### 3.1 Demographics and Clinicopathologic Characteristics

Patient files of 125 patients who underwent tumor resection and flap reconstruction between 2019 and 2022 were screened. Defect repair using a pedicled flap was performed in 13 patients. These patients were excluded from the study. Furthermore, two patients with missing information on complications were excluded, as the risk factors were also unknown. Ultimately, 110 free flap cases were included in the analysis, all in the context of oncological resection.

The median age at surgery was 66 years (range, 37–93 years), and most patients were male (male to female ratio, 2,5:1). The detailed demographic, oncological, medical, and surgical risk data are summarized in Table 1. Looking at classic oncologic head and neck risk factors, 40% of patients had alcohol abuse, and 63.6% had nicotine abuse. Most patients were slim to cachectic (82.7%), of whom 21.8% had a low albumin level of ≤ 33 g/l. Anticoagulation was the primary perioperative treatment. The types of anticoagulation were preoperative in 32.7% (antiplatelet agents, or phenprocoumon, or drugs directly inhibiting blood clotting factors), intraoperative heparin bolus (90%), and postoperative low‐dose heparin in therapeutic or prophylactic dosages (82.7% or 15.5%).

**Table 1:** Demographics and clinicopathologic characteristics.

	*N* a	%
Gender		
Male	79	71.8
Female	31	28.2
Age		
< 55 years	15	13.6
≥ 56 years	95	86.4
pT		
pT0‐2	56	50.9
pT3‐4	44	40
pN		
pN0‐1	70	63.6
pN2‐3	29	26.4
Alcohol		
Yes	44	40
No	47	42.7
Smoking		
Yes	70	63.6
No	40	36.4
Hypertension		
Yes	58	52.7
No	52	47.3
PAVD		
Yes	7	6.36
No	103	93.6
Obesity		
Yes	19	17.3
No	91	82.7
CVE		
Yes	14	12.7
No	96	87.3
DM		
Yes	13	11.8
No	97	88.2
Albumin g/l		
≤ 33	24	21.8
33.1–48	77	70
Defect site		
Mucosa	94	85.5
Skin	16	14.5
preOPAC		
Yes	36	32.7
No	74	67.3
intraOP Aspirin		
Yes	7	6.36
No	103	93.6
intraOPHepbolus		
Yes	99	90
No	9	8.18
POPAC		
Prophylactic	17	15.5
Therapeutic	91	82.7
Catecholamines intraop		
Yes	104	94.5
No	6	5.45
Nor.	48	43.6
Akr. + Nor.	50	45.5
Catecholamines postop		
Yes	53	48.2
No	51	46.4

Akr. = Cafedrin hydrochlorid + Theodralin hydrochlorid, CVE = cardiovascular events (embolia, myocardial infarct, apoplex), DM = diabetes mellitus, PAVD = peripheral arterial disease, Mucosa = oral cavity, oropharynx, hypopharynx, larynx, skin = face, neck, Nor. = Norepinephrin, preOPAC = preoperative anticoagulation, POPAC = postoperative anticoagulation.

^a^ not all values add up to *N* 100% because of missing data, *p* < 0.05 significant (*t*‐test).

### 3.2 Complications and Complication Free Survival

**Figure 1:**
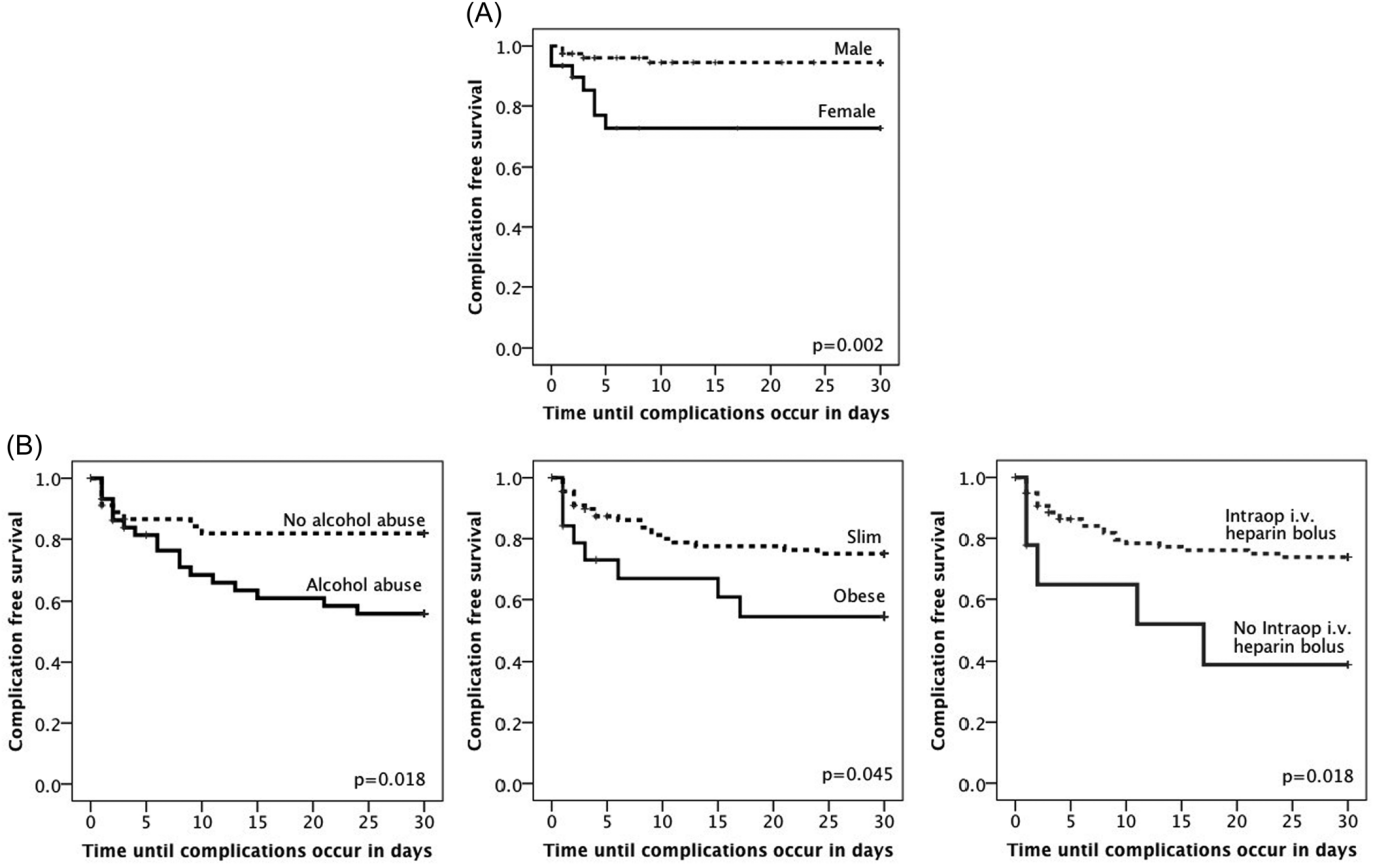
Kaplan‐Meier analysis of 30 day complication free survival of selected variables associated with complications. (A) Female patients (*N* = 31) had a significantly decreased medical complication free survival (CFS) compared to male patients (*N* = 79). (B) Patients with alcohol abuse (*N* = 44), obese patients (*N* = 91), and patients without intraop i.v. heparin bolus had a significantly decreased surgical CSF compared with non‐alcoholics (*N* = 47), slim patients (*N* = 19), and patients without intraop i.v. heparin bolus (*N* = 99).

Complications mainly occurred within the first 5 days after surgery. The one‐, two‐ and 4‐weeks CFS scores were 73.6%, 67.3%, and 63.6%, respectively. The most common surgical complications were wound healing disorders (10.9%), hematoma (10%), and total (7.3%) or partial (1.8%) flap necrosis, mainly due to venous flap thrombosis (4.5%). The most common medical complications were cardiovascular events (5.5%) and pulmonary artery embolism (4.5%). Table 2 presents the variables significantly associated with favorable CFS based on regression analysis, categorized by total, medical, and surgical complications. Key factors affecting surgical and medical CFS are illustrated using Kaplan‐Meier curves in Figure 1. Interestingly, medication, pre‐existing medical illnesses or substance abuse had no influence on medical CFS.

**Table 2:** Complication free survival by relevant demographic, medical and oncologic variables.

		Total		Surgical		Medical	
	*N* a	complications	*p*	complications	*p*	complications	*p*
Gender							
Male	79	70.9%		74.9%		94.6%	
Female	31	45.2%	0.04*	62.5%	0.132	72.7%	0.002*
Age							
< 55 years	15	73.3%		73.3%		100%	
≥ 56 years	95	62.1%	0.272	71.4%	0.677	87%	0.149
pT							
pT0‐2	56	58.9%		71.4%		80.7%	
pT3‐4	44	65.9%	0.450	69.6%	0.799	97.2%	0.016*
pN							
pN0‐1	70	64.3%		70.1%		91.4%	
pN2‐3	29	55.2%	0.459	70.3%	0.742	78.7%	0.063^(*)^
Alcohol							
Yes	44	50.0%		55.9%		89.8%	
No	47	74.5%	0.034*	81.9%	0.018*	88.8%	0.831
Smoking							
Yes	70	62.9%		69.5%		90.8%	
No	40	65%	0.931	75.2%	0.597	86.1%	0.503
Hypertension							
Yes	58	56.9%		67.8%		86.0%	
No	52	71.2%	0.094	75.7%	0.298	91.9%	0.366
PAVD							
Yes	7	85.7%		85.7%		100%	
No	103	62.1%	0.244	70.5%	0.421	88.2%	0.363
Obesity							
Yes	19	47.4%		54.7%		92.1%	
No	91	67%	0.087^(*)^	75%	0.059^(*)^	88.2%	0.543
CVE							
Yes	14	64.3%		69.2%		100%	
No	96	63.5%	0.981	71.9%	0.843	87.5%	0.207
DM							
Yes	13	61.5%		80%		84.6%	
No	97	63.9%	0.777	70.6%	0.464	89.5%	0.457
Albumin							
≤ 33	24	54.2%		57.2%		94.7%	
33.1–48	77	66.2%	0.304	74.6%	0.088^(*)^	87.6%	0.313
Defect site							
Mucosa	94	66.6%		72.8%		90.5%	
Skin	16	50%	0.078^(*)^	63.5%	0.299	81.3%	0.099^(*)^
preOPAC							
Yes	36	52.8%		61.5%		86.7%	
No	74	68.9%	0.072^(*)^	76.5%	0.060^(*)^	90%	0.676
intraOP Aspirin							
Yes	7	57.1%		57.1%		100%	
No	103	64.1%	0.678	72.6%	0.299	88.3%	0.396
intraOPHepbolus							
Yes	99	65.7%		73.8%		88.9%	
No	9	33.3%	0.031*	38.9%	0.018*	100%	0.766
POPAC							
Prophylactic	17	70.6%		70.6%		100%	
Therapeutic	91	62.6%	0.513	72.3%	0.869	86.6%	0.138
Catecholamines postop							
Yes	53	63.5%		71.1%		88.4%	
No	51	66.7%	0.921	80%	0.65	100%	0.427

Akr. = Cafedrinhydrochlorid + Theodralinhydrochlorid, CVE = cardiovascular events (embolia, myocardinfarct, apoplex), DM = diabetis mellitus, Mucosa = oral cavity, oropharynx, hypopharynx, larynx, Skin = face, neck, Nor. + Norepinephrin, PAVD = peripheral arterial disease, POPAC = postoperative anticoagulation, preOPAC = preoperative anticoagulation.

^a^ not all values add up to N 100% because of missing data, *p < 0.05 significant, ^(*)^ *p* < 0.1 trend.

### 3.3 Preventative Factors for Complications

Preventative factors and risks were determined based on the statistical complication analysis. Dichotomizing demographic, medical and oncological characteristics revealed statistically significant differences in CFS (Table 3). Among these characteristics, the greatest absolute differences in percent CFS were observed between the heparin bolus treatment groups (34.9%, *p* = 0.018) and alcohol abuse and no alcohol abuse groups (26%, *p* = 0.018), followed by the gender groups (25.7%, *p* = 0.04) and slim and overweight body constitution groups (20.3%%, *p* = 0.059). Although still marginal significant preoperative anticoagulation groups (15%, *p* = 0.06) and pN status (12.7%, *p* = 0.063) had an impact on CFS among the analyzed variables.

**Table 3:** Prognostic categorization by univariate analysis.

Preventative factors	CFS	Hazardous factors	CFS	*p*	Difference	Type of complications
Male gender	70.9%	Female gender	45.2%	0.04	25.7%	Medical and Surgical
pN0‐1	91.4%	pN3‐4	78.7%	0.063	12.7%	Medical
Alcohol abuse	81.9%	No alcohol abuse	55.9%	0.018	26%	Surgical
Slim body	75%	Overweight	54.7%	0.059	20.3%	Surgical
High albumin	74.6%	Low albumin	57.2%	0.088	17.4%	Surgical
Mucosa defect	66.6%	Skin defect	50%	0.078	16.6%	Medical and Surgical
No Preoperativ anticoagulation	76.5%	Preoperative anticoagulation	61.5%	0.060	15%	Surgical
Intraoperative heparin bolus	73.8%	No intraoperative heparin bolus	38.9%	0.018	34.9%	Surgical

These factors, along with age, nicotine abuse, peripheral artery disease, cardio vascular events, diabetes mellitus, T status, anticoagulation, and catecholamine treatment, were initially included in a univariate Cox regression analysis. While many variables showed statistical significance, multivariable analysis identified gender, alcohol consumption status, body fat status, defect site, preoperative anticoagulation, and intraoperative heparin bolus as independent prognostic factors (Table 4). Male gender shows the greatest protective effect against medical complications compared to female gender (hazard ratio (HR): 9.365, 95% confidence interval CI: 2.619–33.484). Likewise, the absence of alcohol abuse in the patient's history provides more than three times the protection against complications compared to alcohol abuse (HR: 3.454, 95% CI: 1.420–8.406). Although nearly equivalent in effect, low N status (N0‐1), slim body constitution and no need for preoperative anticoagulation contributed to more than two times the prevention of complications compared to N2‐3 cases (HR: 2.399, 95% CI: 1.312–4.388), obesity (HR: 2.121, 95% CI: 0.882–5.098) and preoperative anticoagulation (HR: 2.507, 95% CI: 1.050–5.896). Lastly, early T stage (T1‐2) was associated with better medical CFS outcomes (HR: 0.305, 95% CI: 0.108–0.858).

**Table 4:** Univariate and multivariable cox regression analysis for complications.

	30‐Day CFS			
Total complications	Univariate		Multivarible	
	HR (95% CI)	*p* Value	HR (95% CI)	*p* Value
Age				
< 55 years	Ref			
≥ 55 years	1.753 (0.623–4.930)	0.287		
Gender				
Male	Ref			
Female	2.422 (1.291–4.543)	0.006*	3.191 (1.519–6.706)	0.002*
Alcohol abuse				
Yes	0.482 (0.238–0.976)	0.043*	3.454 (1.420–8.406)	
No	Ref		Ref	0.006*
Hypertension				
Yes	1.7 (0.896–3.228)	0.105		
No	Ref			
Nikotin				
Yes	1.029 (0.537–1.970)	0.932		
No	Ref			
pAVK				
Yes	Ref			
No	0.332 (0.46–2.418)	0.277		
Obesity				
Yes	1.828 (0.893–3.742)		2.121 (0.882–5.098)	
No	Ref	0.099	Ref	0.162
CVE				
Yes	Ref			
No	0.989 (0.387–2.525)	0.982		
DM				
Yes	Ref			
No	1.142 (0.447–2.916)	0.781		
Albumin				
< 33	0.697 (0.344–1.411)	0.315		
≥ 33	Ref			
pT				
pT0‐2	Ref			
pT3‐4	0.783 (0.408–1.5)	0.460		
pN				
pN0‐1	Ref			
pN2‐3	1.132 (0.81–1.582)	0.469		
Defect site				
Mucosa	0.511 (0.235–1.110)	0.09(*)	0.315 (0.119–0.832)	0.018*
Skin	Ref		Ref	
preOPAC				
Yes	1.747 (0.932–3.276)		2.411 (1.190–4.886)	
No	Ref	0.082(*)	Ref	0.015*
intraOP Aspirin				
Yes	1.276 (0.393–4.141)	0.685		
No	Ref			
intraOP Hepbolus				
Yes	0.405 (0.169–0.966)		0.864 (0.296–2.525)	
No	Ref	0.041*	Ref	0.789
Catecholamines intraop				
Yes	Ref			
No	1.073 (0.259–4.448)	0.923		
Hepperfusor postop				
Yes	2.23 (0.685–7.257)	0.183		
No	Ref			
Anticoagulation postop				
Low dose heparin	Ref			
Hepperfusor	1.358 (0.531–3.474)	0.523		
Low dose hep anticoagulation postop				
Prophylactic	Ref			
Therapeutic	2.256 (0.693–7.343)	0.177		
Surgical complications				
Age				
< 55 years	Ref			
≥ 55 years	1.248 (0.434–3.592)	0.681		
Gender				
Male	Ref			
Female	1.778 (0.824–3.833)	0.142		
Alcohol abuse				
Yes	2.598 (1.128–5.984)	0.025*	2.73 (1.174–7.526)	0.021*
No	Ref			
Hypertension				
Yes	1.471 (0.702–3.083)	0.307		
No	Ref			
Nikotin				
Yes	1.233 (0.561–2.708)	0.602		
No	Ref			
pAVK				
Yes	Ref			
No	0.455 (0.062–3.342)	0.439		
Obesity				
Yes	2.129 (0.942–4.813)	0.069^(*)^		
No	Ref			
CVE				
Yes	1.111 (0.386–3.192)	0.845		
No	Ref			
DM				
Yes	Ref			
No	0.592 (0.141–2.491)	0.475		
Albumin				
< 33	0.52 (0.240–1.128)	0.098		
≥ 33				
pT				
pT0‐2	Ref			
pT3‐4	1.050 (0.720–1.531)	0.802		
pN				
pN0‐1	0.931 (0.606–1.432)	0.746		
pN2‐3	Ref			
Defect site				
Mucosa	0.607 (0.231–1.592)	0.310		
Skin	Ref			
preOPAC				
Yes	1.977 (0.949–4.119)	0.069^(*)^	2.507 (1.050–5.896)	0.039*
No	Ref			
intraOP Aspirin				
Yes	1.850 (0.560–6.116)	0.313		
No				
intraOP Hepbolus				
Yes	0.335 (0.128–0.881)	0.027*		
No	Ref			
Catecholamines intraop				
Yes	Ref			
No	1.572 (0.214–11.554)	0.657		
Hepperfusor postop				
Yes	3.368 (1.013–11.194)	0.048*		
No	Ref			
Anticoagulation postop				
Low dose heparin	Ref			
Hepperfusor	3.416 (1.028–11.353)	0.045*		
Low dose hep anticoagulation postop				
Prophylactic	Ref			
Therapeutic	0.922 (0.351–2.427)	0.870		
Medical complications				
Age				
< 55 years	Ref			
≥ 55 years	26.285 (0.023–29783.7)	0.362		
Gender				
Male	Ref			
Female	5.511 (1.607–18.891)	0.007*	9.365 (2.619–33.484)	0.001*
Alcohol abuse				
Yes	Ref			
No	0.867 (0.233–3.233)	0.867		
Hypertension				
Yes	1.747 (0.511–5.976)	0.374		
No	Ref			
Nikotin				
Yes	Ref			
No	0.669 (0.204–2.194)	0.669		
pAVK				
Yes	Ref			
No	0.045 (0.0–1289.9)	0.553		
Obesity				
Yes	Ref			
No	0.535 (0.068–4.180)	0.535		
CVE				
Yes	Ref			
No	0.041 (0–98.21)	0.42		
DM				
Yes	1.771 (0.383–8.2)	0.465		
No	Ref			
Albumin				
< 33	Ref			
≥ 33	2.765 (0.35–21.829)	0.335		
pT				
pT0‐2	0.35 (0.125–0.978)	0.045*	0.305 (0.108–0.858)	0.024*
pT3‐4	Ref			
pN				
pN0‐1	Ref			
pN2‐3	1.707 (0.943–3.09)	0.077^(*)^	2.399 (1.312–4.388)	0.005*
Defect site				
Mucosa	0.344 (0.091–1.303)	0.116		
Skin	Ref			
preOPAC				
Yes	1.298 (0.379–4.441)	0.678		
No	Ref			
intraOP Aspirin				
Yes	Ref			
No	0.045 (0–2503.2)	0.045*		
intraOP Hepbolus				
Yes	0.733 (0.094–5.748)	0.768		
No	Ref			
Catecholamines intraop				
Yes	Ref			
No	21.845 (0–2413225.4)	0.603		
Hepperfusor postop				
Yes	Ref			
No	0.047 (0–43118.3)	0.662		
Anticoagulation postop				
Low dose heparin	Ref			
Hepperfusor	26.57 (0.27–26039.9)	0.351		
Low dose hep anticoagulation postop				
Prophylactic	Ref			
Therapeutic	0.047 (0–45164.7)	0.663		

Abbreviation: CI = confidence interval, HR = hazard ratio.

*p ≤ .05 = significant, (*)*p* < 0.1 = trend.

### 3.4 Association of Individual Complications With Protective and Risk Factors

**Figure 2:**
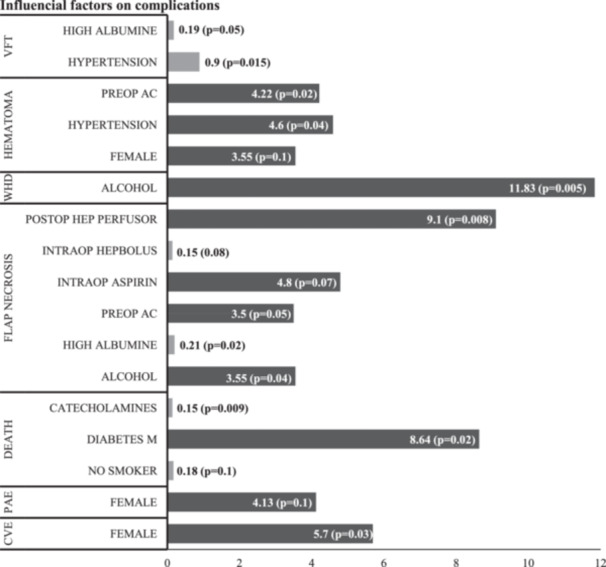
Influencial factors on complications. Odds ratios of influential factors (horizontal) for selected complications (vertical) are shown as bars. Grey: protective factors for complications. Black: risk factors. *p* < 0.05, significant; *p* = 0.06–0.1, trend (Chi Square). CVE = cardiovascular event, PAE = pulmonary artery embolism, preOP AC = preoperative anticoagulation, postOP Hep Perfusor = postoperative heparin perfusion therapy, WHD = wound healing disorder, preOP AC = preoperative anticoagulation, VFT = venous flap thrombus, intraOP Hepbolus = intraoperative heparin bolus.

Factors preventing or contributing directly to certain surgical complications such as venous flap thrombosis, flap necrosis, hematoma, wound healing disorder associated with risk factors were identified and are specified in Figure 2. This figure also takes into account helpful and risk‐associated features for special medical complications such as pulmonary artery embolism, cardiovascular events, and death. A significant reduction in venous flap thrombosis and flap necrosis was observed in patients with high albumin levels (OR 0.21, *p* = 0.02), administration of an intraoperative heparin bolus (OR 0.15, *p* = 0.08), and hypertension (OR 0.9, *p* = 0.015). The highest risk for wound healing disorders was associated with alcohol abuse (OR 11.83, *p* = 0.005). Furthermore, medically ill patients requiring continuous intravenous postoperative therapy with a heparin also demonstrated a correlation with flap necrosis (OR 9.1, *p* = 0.008). Intraoperative catecholamine (norepinephrine) administration and no history of smoking had positive effects on the mortality rate (OR 0.15, *p* = 0.009 and OR 0.18, *p* = 0.1, respectively).

Supporting Table 1 additionally shows that male gender, alcohol abstinence, a slim body constitution, and intraoperative heparin administration are significant protective factors against medical and surgical complications using the odds ratios.

## 4 Discussion

Although microvascular reconstruction is continuously improving, complications occur in up to 14% [10]. Wang et al. describe the largest proportion of complications to be surgical, occurring in up to 11% of cases, while medical complications occur in less than 4% [11]. Treatment prognosis decreases with an increase in patient morbidity. Furthermore, extended hospitalization results in higher public health costs [12]. Therefore, preventive factors should be fully utilized.

The age and gender distribution of the present study is comparable to that of frequently published oncological head and neck patient populations, with a noticeably higher number of male cancer patients [2, 7, 13]. A reason for the gender gap could be related to oncological risk factors such as smoking and alcohol abuse, which have been more common in men, but are recently becoming more common in the female population as well [14]. Poor body constitution due to an underdiagnosed cardiovascular risk profile is a gender‐related aspect that favors males and negatively affects females [15]. Therefore, male gender appears to be an independent preventing key factor against medical complications. However, in reality, these complications are more likely attributed to cardiovascular risk factors rather than to female gender itself. Our gender comparison yielded a significantly lower risk of total and medical complication rate for men. Subgroup analysis showed a significantly higher correlation between hematoma, pulmonary artery embolism, cardiovascular events and women. This is consistent with other studies that evaluated the female gender as an independent risk factor [16].

In our cohort, most patients (2/3) were heavy smokers, and approximately 50% were active alcoholics. The majority of head and neck cancer patients suffer from alcohol and tobacco abuse [17]. Absence of alcohol abuse is as a significant characteristic preventing surgical complications, especially impaired wound healing and flap necrosis. Crawly et al. came to a similar conclusion, postulating a perioperative strategy for alcohol abuse patients, considering alcohol abstinence at least 1 week before surgery, and close follow‐ups with a physician during this time. If not feasible, these authors discussed alternative reconstructive approaches, such as pedicled flaps [18]. Furthermore, smoking is commonly referred to as a risk factor for surgical complications [19, 20]. Interestingly, we did not find smoking to be significantly correlated with any specific surgical complication. But non‐smokers had a higher overall survival rate and less medical complications. In a meta‐analysis, Garip et al. confirmed that there is no correlation between smoking and flap failure, surgical site infection, or fistula formation [21].

Microvascular anastomosis is a critical step in free flap surgery. Perioperative venous flap thrombosis is the main cause of free flap failure and occurs most commonly during the first 48 h [12]. Our data showed that serum albumin levels above 33 g/dl significantly decreased the risk of venous flap thrombosis, marking high albumin as a protective factor against flap necrosis. This is in strong agreement with other studies in which low serum albumin levels, for example, due to malnutrition, increased the risk of flap failure fourfold [22]. The correlation between low albumin levels and the risk of thrombosis has also been confirmed in orthopedic surgery as a low serum albumin level has been shown to be an independent risk factor for deep vein thrombosis in patients undergoing total joint arthroplasty [23]. This raises the question of whether preoperative albumin supplementation can prevent complications. It has been reported that both albumin supplementation and the optimization of nutritional status, for which albumin concentration is a marker, offer benefits for patients undergoing microvascular reconstruction. In a single‐center retrospective study involving over 300 patients, Xu et al. found that patients receiving albumin supplementation had a lower rate of local complications following microvascular head and neck reconstruction. This resulted in a shorter postoperative hospital stay for these patients [24]. Additionally, serum albumin supplementation can reduce the need for perioperative crystalloid infusion, a finding that may be related to albumin's role in maintaining colloid osmotic pressure, thereby supporting more stable hemodynamics [25]. High albumin levels are an indicator of adequate nutrition. Good nutrition is associated with decreased mortality, less flap failure, and lower rate of other major complications following head and neck free flap surgery [26, 27]. Optimizing preoperative nutritional status before free flap procedures may reduce morbidity and mortality, making a high serum albumin level another key factor for successful flap surgery.

Good nutrition does not equate to obesity, as obesity itself is associated with an increased risk of venous flap thrombosis in patients undergoing microvascular head and neck reconstruction [28]. A body mass index (BMI) of less than 28 is correlated with fewer venous thromboembolic events and a shorter hospital stay [29]. Our findings also demonstrate that a normal, slim body constitution reduces the surgical complication rate by half, making a BMI of less than 30 an additional important factor in preventing flap loss and other surgical complications.

Interestingly, hypertension also functions as a protective factor, significantly decreasing the risk of venous flap thrombosis in this study. It leads to faster arterial blood flow in the recipient vessels, which is transmitted to the venous pedicle of the free flap, decreasing the risk of thrombosis. Although this correlation was already shown by Crawley et al. we also found hypertension to be significantly correlated with hematoma. The pathophysiology seems plausible and has been confirmed in our evaluation as well as in several other studies [7, 12, 18, 28, 29, 30].

The same applies to the preoperative anticoagulation therapy. Not being on preoperative anticoagulation therapy has a highly preventive effect against complications such as hematoma or flap necrosis. According to our data, it reduces the rate of these complications by more than half. Other authors have also found that preoperative anticoagulation with acetylsalicylic acid or high‐dose phenprocoumon is significantly correlated with flap necrosis, potentially causing compression of the flap pedicle due to an increased risk of hematoma [31].

In contrast, intraoperative heparin bolus (2.500 to 5.000 IE) appeared to be a very important protective factor against flap necrosis. In a systematic review, Kotamarti et al describe preemptive therapeutic anticoagulation in hypercoagulable patients to improve outcomes in free tissue transfer, though with an increased risk of bleeding [32]. In our data, however, there was no correlation with an increased risk of bleeding. Continuing low‐dose heparin as antithrombotic prophylaxis did not lead to bleeding complications, confirming the results of our previous study [33]. This result is also in line with Tamse et al. who evaluated different regimes of antithrombotic prophylaxis in free flap patients all without significant differences in postoperative bleeding [34]. The continuation of high‐dose continuous intravenous heparin therapy was significantly correlated with flap problems. Patients in need of a postsurgical intravenous therapy usually also have other factors that lead to poor body constitution and flap loss. This aligns with other studies that also identified intraoperative heparin boluses as beneficial; however, high continuous doses were associated with an increased risk of complications [35, 36]. The same is true for prolonged administration of acetylsalicylic acid that leads to a significant increase in complication rate [36].

As another independent risk factor, we found diabetes mellitus Type II, which was strongly correlated with the mortality rate (OR 8.64). Hypoglycemia, easily caused by usual preoperative fasting, increases morbidity and mortality in diabetic patients as well as the length of intensive care and hospital stay. According to very detailed Cochrane review, it is not proven to what extent intensified blood sugar regulation can really reduce perioperative mortality in diabetes mellitus patients [37]. However, based on our results, which show a significant correlation between type II diabetes mellitus and increased mortality, we believe that at least an intensified perioperative and postoperative attention to diabetes mellitus patients could help reduce mortality in this group. Previously, diabetes mellitus type II was also shown to negatively influence the outcome of flap surgery [38].

Intraoperative catecholamine treatment has a positive effect on patient survival compared to patients who did not receive catecholamines during surgery. Despite its vasoconstrictive effect, we did not find any harmful effects on flap perfusion. In contrast, the intraoperative use of dobutamine and norepinephrine has been reported to improve free‐flap blood flow [39, 40].

In general, patients with cardiac preconditions have an increased risk of complications and overall morbidity after noncardiac surgery [41]. In contrast, we did not find a higher perioperative complication rate in patients with a positive cardiovascular history. Peripheral arterial disease (PAVD) as a vascular precondition showed no significant correlation with surgical or medical complications. This has been documented previously in a study comparing flap perfusion in patients with vascular comorbidities such as PAVD, arterial hypertension, atherosclerotic vascular disease, and patients without vascular comorbidities [42].

This study has some limitations. The main limitation is its retrospective, monocentric design. Furthermore, the cohort size restricts the generalizability of the conclusions. Furthermore, the analysis did not include factors such as surgical duration, type of anastomosis, and surgeon variability. However, despite these limitations, this study identifies key preventive factors against complications to improve free flap surgery outcomes.

## 5 Conclusion

In conclusion, considering the appropriate preventive measures can help avoid many complications. This aids in the overall selection of patients and the choice of transplants. This study has identified characteristics that help determine who requires special preconditioning, which intraoperative treatments are necessary, and who needs dedicated postoperative monitoring.

The most important factors in preventing perioperative surgical complications in free flap surgery include nutritional status correlated with adequate blood albumin levels, intraoperative heparin administration, maintaining adequate blood pressure, and the absence of alcohol abuse.

The factors that help prevent medical complications and improve survival include a nonsmoking status and intraoperative catecholamine treatment. However, the absence of a need for high‐dose heparin treatment, male gender, normoglycemia, and overall metabolic health are strongly associated with fewer medical complications and lower mortality. Vascular diseases, such as PAVD, and age over 60 years showed no correlation with increased complication rates. Therefore, flap planning is considered safe for these patients.

In summary, the following pre‐, intra‐, and postoperative recommendations should be considered. *Preoperatively*, female patients should be screened for undiagnosed cardiovascular risks. In patients with diabetes mellitus, blood sugar levels should be stabilized, and preoperative fasting should be avoided. For malnourished patients and those with low albumin levels, nutritional status should be optimized in a timely manner before surgery, if possible.

*Intraoperatively*, the following measures have been shown to significantly improve flap and patient survival: intravenous heparin bolus administration, catecholamine administration, and maintaining stable blood sugar levels.

*Postoperatively*, several important aspects must be carefully considered. In particular, the following groups should be closely monitored to prevent medical complications: female patients, those with high TNM status, and diabetics. To detect surgical complications early, patients with active alcoholism, hypertension, and low albumin levels should undergo close monitoring of wound healing and flap condition. Additionally, albumin supplementation may be considered in cases of hypoalbuminemia to reduce the risk of surgical complications.

## Conflicts of Interest

The authors declare no conflicts of interest.

## Synopsis

This recent study evaluates complication parameters in free flap head and neck surgery patients in a retrospective design to find key preventive factors to decrease the risk of adverse events. Optimizing nutritional status and albumin levels, administering intraoperative heparin and catecholamines, and abstaining from alcohol were discussed as potential helpful parameters.

## Supporting information


**Table S1:** Demographic and pathophysiologic characteristics associated with complications.

## Data Availability

The data that support the findings of this study are available from the corresponding author upon reasonable request.

## References

[1] A. Al‐lami , A. Al‐Asfoor , A. A. Khoo , N. G. Patel , R. D. Price , and A. J. Durrani , “Free Tissue Transfer in Head and Neck Reconstruction: 100 Consecutive Cases,” B‐ENT 11 (2015): 51–56. 26513948

[2] E. T. Carniol , E. Marchiano , J. S. Brady , et al., “Head and Neck Microvascular Free Flap Reconstruction: An Analysis of Unplanned Readmissions,” Laryngoscope 127 (2017): 325–330. 27140439 10.1002/lary.26039

[3] C. Copelli , K. Tewfik , L. Cassano , et al., “Management of Free Flap Failure in Head and Neck Surgery,” Acta Otorhinolaryngologica Italica 37 (2017): 387–392. 29165433 10.14639/0392-100X-1376 PMC5720866

[4] M. Koch , K. Jalyzada , P. Grundtner , et al., “Treatment of the Donor Site of Free Radial Flaps: Vacuum Sealing Versus Conventional Wound Care,” Acta Oto‐Laryngologica 137 (2017): 1301–1306. 28754077 10.1080/00016489.2017.1357190

[5] S. F. Politano , D. Balchander , C. I. Cabrera , et al., “Impact of Intraoperative Ischemia Time on Acute Complications of Head and Neck Microvascular Free Tissue Transfer: A Systematic Review and Meta‐Analysis,” American Journal of Otolaryngology 43 (2022): 103467. 35429849 10.1016/j.amjoto.2022.103467

[6] A. Walia , J. J. Lee , R. S. Jackson , et al., “Management of Flap Failure After Head and Neck Reconstruction: A Systematic Review and Meta‐Analysis,” Otolaryngology–Head and Neck Surgery 167 (2022): 224–235. 34491852 10.1177/01945998211044683 PMC8972962

[7] W. Zhou , W. B. Zhang , Y. Yu , et al., “Risk Factors for Free Flap Failure: A Retrospective Analysis of 881 Free Flaps for Head and Neck Defect Reconstruction,” International Journal of Oral and Maxillofacial Surgery 46 (2017): 941–945. 28416356 10.1016/j.ijom.2017.03.023

[8] J. C. Dort , D. G. Farwell , M. Findlay , et al., “Optimal Perioperative Care in Major Head and Neck Cancer Surgery With Free Flap Reconstruction: A Consensus Review and Recommendations From the Enhanced Recovery After Surgery Society,” JAMA Otolaryngology–Head & Neck Surgery 143 (2017): 292–303. 27737447 10.1001/jamaoto.2016.2981

[9] A. Y. Shen , S. Lonie , K. Lim , H. Farthing , D. J. Hunter‐Smith , and W. M. Rozen , “Free Flap Monitoring, Salvage, and Failure Timing: A Systematic Review,” Journal of Reconstructive Microsurgery 37 (2021): 300–308. 33395711 10.1055/s-0040-1722182

[10] D. T. Bui , P. G. Cordeiro , Q. Y. Hu , J. J. Disa , A. Pusic , and B. J. Mehrara , “Free Flap Reexploration: Indications, Treatment, and Outcomes in 1193 Free Flaps,” Plastic and Reconstructive Surgery 119 (2007): 2092–2100. 17519706 10.1097/01.prs.0000260598.24376.e1

[11] C. Wang , N. Liufu , F. Ji , Z. Han , Z. Liu , and M. Cao , “Risk Factors Associated With Postoperative Complications Following Free Flap Reconstruction of Head and Neck Defects,” Journal of Stomatology, Oral and Maxillofacial Surgery 123 (2022): e894–e898. 34971838 10.1016/j.jormas.2021.12.013

[12] P. Sanati‐Mehrizy , B. B. Massenburg , J. M. Rozehnal , M. J. Ingargiola , J. Hernandez Rosa , and P. J. Taub , “Risk Factors Leading to Free Flap Failure: Analysis From the National Surgical Quality Improvement Program Database,” Journal of Craniofacial Surgery 27 (2016): 1956–1964. 28005734 10.1097/SCS.0000000000003026

[13] B. B. Yarlagadda , D. G. Deschler , D. L. Rich , et al., “Head and Neck Free Flap Surgical Site Infections in the Era of the Surgical Care Improvement Project,” supplement, Head & Neck 38, no. Suppl 1 (2016): E392–E398. 10.1002/hed.24005 25641048

[14] C. Bolego , “Smoking and Gender,” Cardiovascular Research 53 (2002): 568–576. 11861027 10.1016/s0008-6363(01)00520-x

[15] G. Brandrup‐Wognsen , H. Berggren , M. Hartford , A. Hjalmarson , T. Karlsson , and J. Herlitz , “Female Sex Is Associated With Increased Mortality and Morbidity Early, but Not Late, After Coronary Artery Bypass Grafting,” European Heart Journal 17 (1996): 1426–1431. 8880029 10.1093/oxfordjournals.eurheartj.a015078

[16] A. Loupatatzi , S. D. Stavrianos , F. F. Karantonis , et al., “Are Females Predisposed to Complications in Head and Neck Cancer Free Flap Reconstruction?,” Journal of Oral and Maxillofacial Surgery 72 (2014): 178–185. 23850041 10.1016/j.joms.2013.05.013

[17] M. Hashibe , P. Brennan , S. Chuang , et al., “Interaction Between Tobacco and Alcohol Use and the Risk of Head and Neck Cancer: Pooled Analysis in the International Head and Neck Cancer Epidemiology Consortium,” Cancer Epidemiology, Biomarkers & Prevention 18 (2009): 541–550. 10.1158/1055-9965.EPI-08-0347 PMC3051410 19190158

[18] M. B. Crawley , L. Sweeny , P. Ravipati , et al., “Factors Associated With Free Flap Failures in Head and Neck Reconstruction,” Otolaryngology–Head and Neck Surgery 161 (2019): 598–604. 31382816 10.1177/0194599819860809

[19] M. M. Crippen , N. Patel , A. Filimonov , et al., “Association of Smoking Tobacco With Complications in Head and Neck Microvascular Reconstructive Surgery,” JAMA Facial Plastic Surgery 21 (2019): 20–26. 30347003 10.1001/jamafacial.2018.1176 PMC6439727

[20] J. B. Kinsella , C. H. Rassekh , J. A. Hokanson , Z. D. Wassmuth , and K. H. Calhoun , “Smoking Increases Facial Skin Flap Complications,” Annals of Otology, Rhinology, & Laryngology 108 (1999): 139–142. 10030230 10.1177/000348949910800206

[21] M. Garip , J. Van Dessel , L. Grosjean , C. Politis , and M. Bila , “The Impact of Smoking on Surgical Complications After Head and Neck Reconstructive Surgery With a Free Vascularised Tissue Flap: A Systematic Review and Meta‐Analysis,” British Journal of Oral and Maxillofacial Surgery 59 (2021): e79–e98. 33546845 10.1016/j.bjoms.2020.07.020

[22] J. Shum , M. R. Markiewicz , E. Park , et al., “Low Prealbumin Level Is a Risk Factor for Microvascular Free Flap Failure,” Journal of Oral and Maxillofacial Surgery 72 (2014): 169–177. 23911143 10.1016/j.joms.2013.05.022

[23] X. Xiong , T. Li , S. Yu , X. Lu , Q. Mao , and Y. Xiong , “Association Between Low Serum Albumin and Preoperative Deep Vein Thrombosis in Patients Undergoing Total Joint Arthroplasty: A Retrospective Study,” Clinical and Applied Thrombosis/Hemostasis 29 (2023): 10760296231178547. 37248630 10.1177/10760296231178547 PMC10233575

[24] H. Xu , Z. Han , W. Ma , X. Zhu , J. Shi , and D. Lin , “Perioperative Albumin Supplementation Is Associated With Decreased Risk of Complications Following Microvascular Head and Neck Reconstruction,” Journal of Oral and Maxillofacial Surgery 79 (2021): 2155–2161. 34119478 10.1016/j.joms.2021.04.030

[25] C. Polito and G. S. Martin , “Albumin: Physiologic and Clinical Effects on Lung Function,” Minerva Anestesiologica 79 (2013): 1180–1186. 23811622 PMC4226654

[26] I. Herzog , D. Panchal , S. Sikder , et al., “Malnutrition in Head and Neck Free Flap Reconstruction as a Predictor of Adverse Outcomes,” Annals of Plastic Surgery 92 (2024): S251–S254. 38556683 10.1097/SAP.0000000000003868

[27] Y. H. Yen , S. D. Luo , W. C. Chen , et al., “The Value of the Nutritional Indicators in Predicting Free Flap Failure From a Multicentre Database,” Otolaryngology–Head and Neck Surgery 171 (2024): 63–72. 38501382 10.1002/ohn.706

[28] R. E. Berner , W. D. Morain , and J. M. Noe , “Postoperative Hypertension as an Etiological Factor in Hematoma After Rhytidectomy,” Plastic and Reconstructive Surgery 57 (1976): 314–319. 1257337 10.1097/00006534-197603000-00006

[29] P. A. Borggreven , D. J. Kuik , J. J. Quak , R. de Bree , G. B. Snow , and C. R. Leemans , “Comorbid Condition as a Prognostic Factor for Complications in Major Surgery of the Oral Cavity and Oropharynx With Microvascular Soft Tissue Reconstruction,” Head & Neck 25 (2003): 808–815. 12966504 10.1002/hed.10291

[30] C. Vandersteen , O. Dassonville , E. Chamorey , et al., “Impact of Patient Comorbidities on Head and Neck Microvascular Reconstruction. A Report on 423 Cases,” European Archives of Oto‐Rhino‐Laryngology 270 (2013): 1741–1746. 23081673 10.1007/s00405-012-2224-z

[31] B. M. Barton , C. A. Riley , J. C. Fitzpatrick , C. P. Hasney , B. A. Moore , and E. D. McCoul , “Postoperative Anticoagulation After Free Flap Reconstruction for Head and Neck Cancer: A Systematic Review,” Laryngoscope 128 (2018): 412–421. 28581030 10.1002/lary.26703

[32] V. S. Kotamarti , E. Shiah , K. M. Rezak , A. Patel , and J. A. Ricci , “Does Anticoagulation Improve Flap Outcomes in Hypercoagulable Patients? A Systematic Review,” Journal of Reconstructive Microsurgery 36 (2020): 204–212. 31766062 10.1055/s-0039-3400531

[33] M. Sievert , M. Goncalves , R. Tamse , et al., “Postoperative Management of Antithrombotic Medication in Microvascular Head and Neck Reconstruction: A Comparative Analysis of Unfractionated and Low‐Molecular‐Weight Heparin,” European Archives of Oto‐Rhino‐Laryngology 278 (2021): 1567–1575. 32710177 10.1007/s00405-020-06219-w PMC8057982

[34] H. R. Tamse , M. Koch , S. K. Mueller , et al., “Perioperative Anticoagulation in Free Microvascular Flaps‐A Comparison of Different Prophylactic Regimes in Oncologic Reconstructive Surgery,” European Review for Medical and Pharmacological Sciences 28 (2024): 3532–3541. 38856128 10.26355/eurrev_202405_36288

[35] Y. Hataya , K. Matsuo , M. Ishigaki , Y. Imai , and K. Taki , “Retrograde Intra‐Arterial Infusion of Prostaglandin El and Heparin for the No‐Reflow Phenomenon After Oromandibular Reconstruction With a Free Fibular Flap,” Annals of Plastic Surgery 42 (1999): 92–95. 9972725 10.1097/00000637-199901000-00016

[36] R. Rothweiler , V. Gerlach , P. Voss , et al., “Aspirin, Heparin and Ischemia Time in Microvascular Free Flap Surgery ‐ Their Influence and an Optimal Anticoagulation Protocol,” Journal of Stomatology, Oral and Maxillofacial Surgery 123 (2022): e556–e562. 35272089 10.1016/j.jormas.2022.03.001

[37] F. Bellon , I. Solà , G. Gimenez‐Perez , et al., “Perioperative Glycaemic Control for People With Diabetes Undergoing Surgery,” Cochrane Database of Systematic Reviews 8 (2023): 007315. 10.1002/14651858.CD007315.pub3 PMC10392034 37526194

[38] T. H. Chiu , C. K. Tsao , S. N. Chang , J. W. Lin , and J. J. Hwang , “Clinical Consequences of Head and Neck Free‐Flap Reconstructions in the Dm Population,” Scientific Reports 11 (2021): 6034. 33727645 10.1038/s41598-021-85410-3 PMC7966812

[39] K. A. Eley , J. D. Young , and S. R. Watt‐Smith , “Epinephrine, Norepinephrine, Dobutamine, and Dopexamine Effects on Free Flap Skin Blood Flow,” Plastic & Reconstructive Surgery 130 (2012): 564–570. 22929242 10.1097/PRS.0b013e31825dbf73

[40] A. Scholz , S. Pugh , M. Fardy , M. Shafik , and J. E. Hall , “The Effect of Dobutamine on Blood Flow of Free Tissue Transfer Flaps During Head and Neck Reconstructive Surgery*,” Anaesthesia 64 (2009): 1089–1093. 19735400 10.1111/j.1365-2044.2009.06055.x

[41] D. J. Wirthlin and R. P. Cambria , “Surgery‐Specific Considerations in the Cardiac Patient Undergoing Noncardiac Surgery,” Progress in Cardiovascular Diseases 40 (1998): 453–468. 9585377 10.1016/s0033-0620(98)80017-0

[42] M. Ooms , M. Heitzer , P. Winnand , et al., “Impacts of Vascular Comorbidities on Free Flap Perfusion in Microvascular Head and Neck Reconstruction,” European Archives of Oto‐Rhino‐Laryngology 280 (2023): 3375–3382. 36897365 10.1007/s00405-023-07913-1 PMC10220101

