# Supplementary material for: Preventing Free Flap Complications: Key Factors to Keep in Mind
Source: J Surg Oncol. 2025 Aug 27;132(5):945–58. doi: 10.1002/jso.70067 (PMC12501922; doi:10.1002/jso.70067)
Supplement: Supplementary file 1 — Table S1: Demographic and pathophysiologic characteristics associated with complications. [file JSO-132-945-s001.docx]

**Demographic and pathophysiologic characteristics associated with complications**

|  |  |  | Total  complications |  | Surgical complications |  | Medical complications |  |
| --- | --- | --- | --- | --- | --- | --- | --- | --- |
|  |  | N^1^ | Odds Radio (95% CI) | p | Odds Radio (95% CI) | p | Odds Radio (95% CI) | p |
| Gender | |  |  |  |  |  |  |  |
|  | Male | 79 |  |  |  |  |  |  |
|  | Female | 31 | 2.96 (1.25-6.97) | .011* | 1.5 (0.6-3.75) | 0.38 | 5.47 (1.47-20.3) | 0.006** |
| Age | |  |  |  |  |  |  |  |
|  | <55 years | 15 |  |  |  |  |  |  |
|  | ≥56 years | 95 | 1.68 (0.5-5.67) | 0.41 | 0.98 (0.286-3.37) | 0.98 | 1.13 (1.05-1.22) | 0.17 |
| pT |  |  |  |  |  |  |  |  |
|  | pT0-2 | 56 |  |  |  |  |  |  |
|  | pT3-4 | 44 | 0.74 (0.33-1.69) | 0.48 | 1.26 (0.52-3.05) | 0.62 | 0.11 (0.01-0.87) | 0.01* |
| pN | |  |  |  |  |  |  |  |
|  | pN0-1 | 70 |  |  |  |  |  |  |
|  | pN2-3 | 29 | 1.46 (0.6-3.52) | 0.4 | 0.8 (0.29-2.15) | 0.66 | 3.39 (0.94-12.18) | 0.05* |
| Alcohol | |  |  |  |  |  |  |  |
|  | Yes | 44 | 2.92 (1.21-7.05) | 0.02* | 3.38 (1.28-8.9) | 0.01* | 0.84 (0.21-3.35) | 0.81 |
|  | No | 47 |  |  |  |  |  |  |
| Smoking | |  |  |  |  |  |  |  |
|  | Yes | 70 | 1.1 (0.49-2.47) | 0.82 | 1.38 (0.56-3.41) | 0.49 | 0.66 (0.19-2.3) | 0.51 |
|  | No | 40 |  |  |  |  |  |  |
| Hypertension | |  |  |  |  |  |  |  |
|  | Yes | 58 | 1.87 (0.85-4.13) | 0.12^(*)^ | 1.38 (0.57-3.26) | 0.46 | 1.55 (0.45-5.98) | 0.45 |
|  | No | 52 |  |  |  |  |  |  |
| PAVD | |  |  |  |  |  |  |  |
|  | Yes | 7 |  |  |  |  | 0.89 (0.84-0.96) | 0.37 |
|  | No | 103 | 0.27 (0.32-2.36) | 0.21 | 0.45 (0.05-3.86) | 0.46 |  |  |
| Obesity | |  |  |  |  |  |  |  |
|  | Yes | 19 | 2.26 (0.83-6.15) | 0.11^(*)^ | 2.42 (0.86-6.81) | 0.08^(*)^ |  |  |
|  | No | 91 |  |  |  |  | 0.05 (0.05-3.74) | 0.45 |
| CVE | |  |  |  |  |  |  |  |
|  | Yes | 14 |  |  | 1.14 (0.33-3.95) | 0.84 | 0.89 (0.83-0.95) | 0.2 |
|  | No | 96 | 0.97 (0.3-3.12) | 0.96 |  |  |  |  |
| DM | |  |  |  |  |  |  |  |
|  | Yes | 13 |  |  |  |  | 1.78 (0.34-9.31 | 0.5 |
|  | No | 97 | 1.11 (0.34-3.65) | 0.87 | 0.47 (0.01-2.27) | 0.34 |  |  |
| Albumin g/l | |  |  |  |  |  |  |  |
|  | ≤ 33 | 24 |  |  |  |  |  |  |
|  | 33.1 - 48 | 77 | 0.6 (0.24-1.53) | 0.29 | 0.43 (0.162-1.12) | 0.08^(*)^ | 3.04 (0.37-25.35) | 0.29 |
| Defect site | |  |  |  |  |  |  |  |
|  | mucosa | 94 | 0.52 | 0.22 |  |  | 0.4 (0.1-1.72) | 0.21 |
|  | Skin | 16 |  |  | 0.75 (0.24-2.39) | 0.64 |  |  |
| preOPAC | |  |  |  |  |  |  |  |
|  | Yes | 36 | 1.86 (0.88-4.5) | 0.1^(*)^ | 2.05 (0.85-4.92) | 0.1^(*)^ | 1.2 (0.33-4.38) | 0.8 |
|  | No | 74 |  |  |  |  |  |  |
| intraOP Aspirin | |  |  |  |  |  |  |  |
|  | Yes | 7 | 1.34 (0.28-6.3) | 0.72 | 2.22 (0.47-10.59) | 0.31 | 0.89 (0.84-0.96) | 0.37 |
|  | No | 103 |  |  |  |  |  |  |
| intraOPHepbolus | |  |  |  |  |  |  |  |
|  | Yes | 99 | 0.26 (0.06-1.11) | 0.55^(*)^ | 0.26 (0.06-1.03) | 0.04* | 0.9 (0.1-7.95) | 0.92 |
|  | No | 9 |  |  |  |  |  |  |
| POPAC | |  |  |  |  |  |  |  |
|  | prophylactic | 17 |  |  | 0.81 (0.26-2.55) | 0.72 |  |  |
|  | therapeutic | 91 | 1.43 (0.46-4.42) | 0.54 |  |  | 1.14 (1.05-1.23) | 0.13 |
| Catecholamines intraop | | |  |  |  |  |  |  |
|  | Yes | 104 | 1.15 (0.2-6.59) | 0.88 | 1.84 (0.2-16.5) | 0.58 | 1.12 (1.05-1.2) | 0.4 |
|  | No | 6 |  |  |  |  |  |  |
|  | Nor. | 48 |  |  |  |  |  |  |
|  | Akr.+Nor. | 50 | 1.03 (0.45-2.34) | 0.95 | 0.95 (0.34-2.32) | 0.91 | 0.96 (0.258-3.54) | 0.95 |
| Catecholamines postop | | |  |  |  |  |  |  |
|  | Yes | 53 | 0.94 (0.42-2.09) | 0.88 | 0.64 (0.27-1.53) | 0.32 | 2.84 (0.71-11.4) | 0.13 |
|  | No | 51 |  |  |  |  |  |  |

^1^not all values add up to N 100% because of missing data, *p<0.05 significant, (*)p<0.1 trend

PAVD=peripheral arterial disease; CVE=cardiovascular events (embolia, myocardial infarct, apoplex); DM= diabetes mellitus; Mucosa, oral cavity, oropharynx, hypopharynx, larynx; skin, face, neck; preOPAC, preoperative anticoagulation; POPAC, postoperative anticoagulation; Akr. = Cafedrin hydrochlorid + Theodralin hydrochlorid; Nor. +Norepinephrin

Table Supp1
